# Syntheses and Characterization of New Nickel Coordination Polymers with 4,4′-Dipyridylsulfide. Dynamic Rearrangements of One-Dimensional Chains Responding to External Stimuli: Temperature Variation and Guest Releases/Re-Inclusions

**DOI:** 10.3390/ijms11082821

**Published:** 2010-08-02

**Authors:** Mitsuru Kondo, Hideaki Takahashi, Hirotaka Watanabe, Yusuke Shimizu, Katsunori Yamanishi, Makoto Miyazawa, Naoko Nishina, Yutaka Ishida, Hiroyuki Kawaguchi, Fumio Uchida

**Affiliations:** 1 Center for Instrumental Analysis, Shizuoka University, 836 Ohya, Suruga-kul, Shizuoka 422-8529, Japan; 2 Department of Chemistry, Faculty of Science, Shizuoka University, 836 Ohya, Suruga-kul, Shizuoka 422-8529, Japan; E-Mail: asian-dream@nifty.com (H.T.); gibsanusa@gmail.com (H.W.); ky_bi_0117@yahoo.co.jp (K.Y.); m.miyazawa@hang-ichi.com (M.M.); snnishi@ipc.shizuoka.ac.jp (N.N.); 3 Department of Chemistry, Graduate School of Science and Engineering, Tokyo Institute of Technology, 2-12-1 Ookayama, Meguro-ku, Tokyo 152-8551, Japan; E-Mails: yishida@chem.titech.ac.jp (Y.I.); hkawa@chem.titech.ac.jp (H.K.); 4 Hautform Division, Fuji Chemical Co. Ltd. 1683-1880, Nakagaito, Nasubigawa, Nakatsugawa, Gifu 509-9132, Japan

**Keywords:** porous coordination networks, phase transition, dynamic structural change, crystal structure

## Abstract

Crystal structures and dynamic rearrangements of one-dimensional coordination polymers with 4,4′-dipyridylsulfide (dps) have been studied. Reaction of Ni(NO_3_)_2_·6H_2_O with dps in EtOH yielded [Ni(dps)_2_(NO_3_)_2_] ·EtOH (**1**), which had channels filled with guest EtOH molecules among the four Ni(dps)_2_ chains. This coordination polymer reversibly transformed the channel structure responding to temperature variations. Immersion of **1** in *m*-xylene released guest EtOH molecules to yield a guest-free coordination polymer [Ni(dps)_2_(NO_3_)_2_] (**2a**), which was also obtained by treatment of Ni(NO_3_)_2_·6H_2_O with dps in MeOH. On the other hand, removal of the guest molecules from **1** upon heating at 130 °C under reduced pressure produced a guest-free coordination polymer [Ni(dps)_2_(NO_3_)_2_] (**2b**). Although the **2a** and **2b** guest-free coordination polymers have the same formula, they showed differences in the assembled structures of the one-dimensional chains. Exposure of **2b** to EtOH vapor reproduced **1**, while **2a** did not convert to **1** in a similar reaction. Reaction of Ni(NO_3_)_2_·6H_2_O with dps in acetone provided [Ni(dps)(NO_3_)_2_(H_2_O)] ·Me_2_CO (**4**) with no channel structure. When MeOH or acetone was used as a reaction solvent, the [Ni(dps)_2_(NO_3_)_2_] · (guest molecule) type coordination polymer, which was observed in **1,** was not formed. Nevertheless, the reaction of Ni(NO_3_)_2_·6H_2_O with dps in MeOH/acetone mixed solution produced [Ni(dps)_2_(NO_3_)_2_]·0.5(MeOH·acetone) (**5**), which has an isostructural Ni-dps framework to **1**.

## 1. Introduction

Incorporation of dynamic mechanisms into the channel frameworks have attracted intense attention for the development of new functional materials [1–19]. For example, chemical modifications of the frameworks of zeolites have yielded unique functions such as controlled release of the including guest molecules from channels [19]. These functions are important not only for the development of drug delivery systems, but also for highly effective storage, including of guest molecules. On the other hand, many studies have reported that coordination polymers, which are also called metal-organic frameworks (MOFs), with channel structures, afford a variety of infinite network structures [20]. These compounds have been synthesized from metal sources and organic bridging ligands by a self-assembly process. These coordination materials have been considered as a new class of porous materials because they have often shown unique functions, which were not observed in inorganic materials such as zeolites. For example, heterogeneous catalysis [21–24], high gas storages [25–28], and high selective molecular adsorption [1,13,29–32] have been reported. Many porous coordination polymers cannot retain their channel frameworks after the removal of included guest molecules that were incorporated in the channels when they were prepared. In spite of their fragility, some porous coordination polymers have unique adsorption properties, and can selectively re-include organic guest molecules; and reproduce the initial porous framework.

**Scheme 1:**
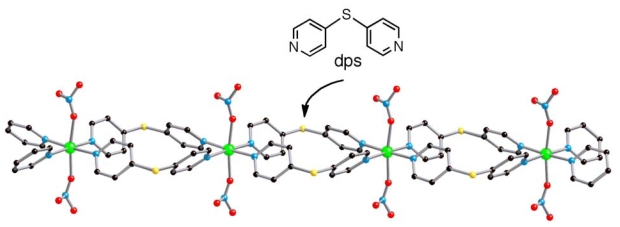
Structure of (**Ni**-**dps2**) chain.

For years we have focused on coordination polymers that change their structures responding to external stimuli such as temperature variation [6] and present organic solvents [33,34]. As a unique example, we reported a new Ni coordination polymer with 4,4′-dipyridylsulfide (dps) in our previous communication [6]. This compound created unique channels, which changed the channel windows responding to temperature variation. The channels below the critical temperature mechanically captured guest EtOH molecules, and then released them above the temperature. This coordination polymer was comprised of one-dimensional frameworks formulated as [Ni(dps)_2_(NO_3_)_2_], which is designated as “(**Ni**-**dps2**) chain” (Scheme 1). This paper describes the unique rearrangement properties of the (**Ni**-**dps2**) chains responding to external stimuli such as temperature variations, and the guest release and re-inclusion.

## 2. Results and Discussion

### 2.1. Overview of the Structural Rearrangement of the Ni-dps System

**Scheme 2:**
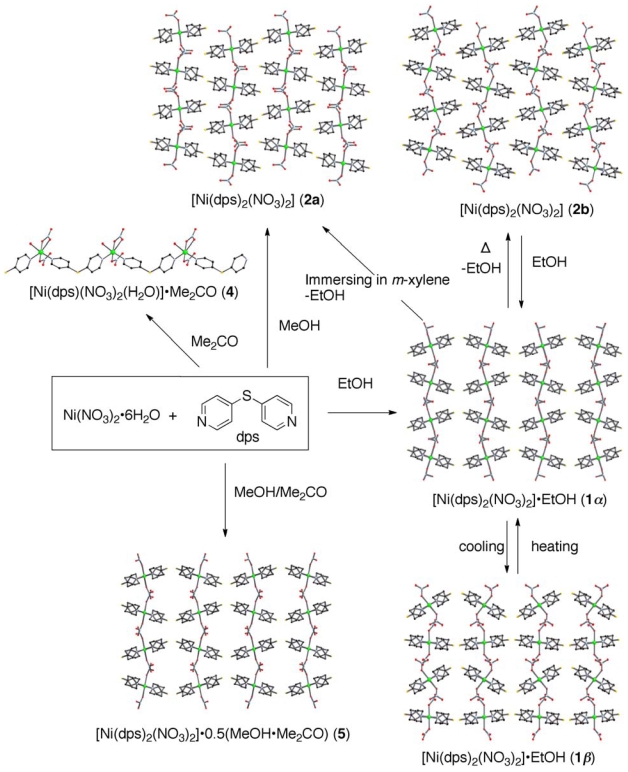
Structures and rearrangement aspects of (**Ni**-**dps2**) chains for the Ni-dps compounds. The structures are drawn along the chains except for **4.**

Scheme 2 summarizes the structures and rearrangement of the (**Ni**-**dps2**) chains in Ni-dps compounds. The views are illustrated along the one-dimensional chain direction except for **4**. Reaction of Ni(NO_3_)_2_·6H_2_O with dps in EtOH or MeOH produced coordination polymers **1** and **2a**, which were constructed by stacks of (**Ni**-**dps2**) chains. **1** had two structural phases that reversibly transformed depending on the temperature about -12 °C. The two structural phases observed above and below the critical temperature were designated as **1*α*** and **1*β*** Immersion of a solid sample of **1** into *m*-xylene released guest EtOH molecules, and converted **1** to **2a**. On the other hand, removal of the guest EtOH molecules from single crystals of **1** on heating at 130 °C under reduced pressure produced dried compound **2b** as a crystalline solid. Although the data quality was poor due to the cracks, **2b** was useful for single crystal X-ray analysis. This means that the guest removal reaction proceeded by the single-crystal-to-single-crystal process [11]. The dried compound **2b** reproduced **1** by exposure to EtOH. Although **2a** and **2b** are guest-free coordination polymers with the same formula, their stacking patterns of (**Ni**-**dps2**) chains are different, meaning that **2b** is an allotrope of **2a**. While **2b** converted to **1** reversibly, **2a** did not convert to **1** in a similar reaction condition.

The reactions of Ni(NO_3_)_2_·6H_2_O with dps in Me_2_CO produced coordination polymer **4**, which was not constructed by (**Ni**-**dps2**) chains, but {Ni(dps)(NO_3_)_2_(H_2_O)}_n_ chains. On the other hand, when the reaction was carried out in MeOH/acetone mixed media, coordination polymer **4**, which had an assembled structure like **1*α***, was obtained.

### 2.2. Crystal Structures of **1α** and **1β**

**Figure 1:**
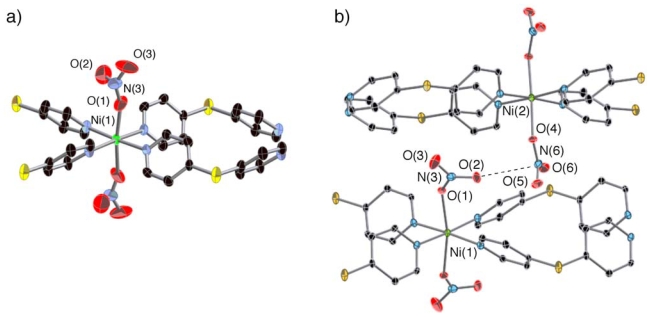
Coordination circumstances of **1*α*** (**a**) and **1*β*** (**b**). Hydrogen atoms are omitted for clarity. **1*β*** contains two crystallographically independent (**Ni**-**dps2**) chains. The nitrate anions in the different chains are connected by electrostatic interactions as shown by dashed line (**b**).

**Figure 2:**
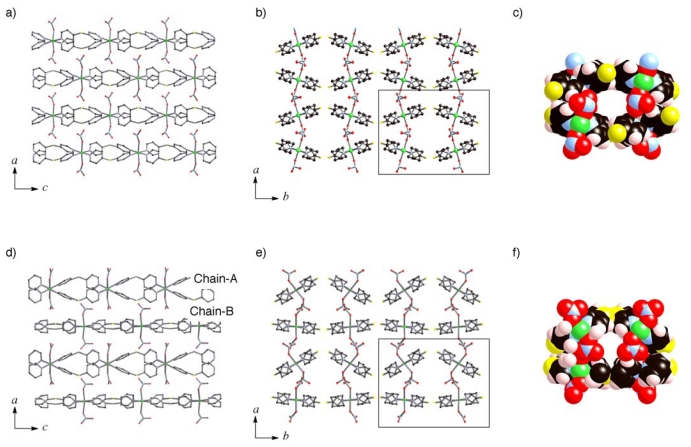
Crystal structures of **1*α*** (**a**-**c**) and **1*β*** (**d**-**f**). Ethanol molecules in the channels of **1*β*** are omitted for clarity. Stacking structures of (**Ni**-**dps2**) chains along the *b* axis (**a**, **c**, **d**, **f**) and *c* axis (**b**, **e**) are exhibited. The channel formed by surrounding four chains is indicated by the rectangles in (**b**) and (**e**). Their channel structures with van der Waals radii are revealed in (**c**) and (**f**). Except for (**c**) and (**f**), the hydrogen atoms are omitted for clarity.

**1** was easily obtained as light-blue crystals by diffusion of dps into the Ni(NO_3_)_2_·6H_2_O in an ethanol solution [6]. Figure 1 and 2 compare the crystal structures of **1*α*** and **1*β*** **1*α*** which is in the structure phase of **1** at room temperature, crystallizes in the centric space group *C*cc2. The structural determination was carried out at 23 °C. The nickel center is based on a distorted octahedron with four pyridine nitrogen atoms and two oxygen atoms from nitrate anions, in which the nitrate anions occupy the axial positions (Figure 1). Each nickel center is bridged by two dps ligands to yield one-dimensional chains with small rhombus cavities (*ca.* 5 × 5 Å) surrounded by two nickel atoms and two dps ligands.

These chains run along the *c* axis. There are two crystallographically equivalent chains with different inclinations to the *a* and *b* axes each, whose tilting angles of NO_3_—Ni—NO_3_ vectors to the *a* axis are about 15° and −15°. These chains alternatively stack along the *a* axis, with the nitrate anions being located above and below the square cavities of the adjacent chains. Among four one-dimensional chains, one-dimensional channels with a compressed octahedral shape (*ca.* 5 × 5 Å) are created along the *c* axis. Although elemental analysis and structural characterization at lower temperature showed that **1** contained one ethanol molecule per nickel atom, the expected electron densities were not observed in the channels of **1*α***, despite that we carried out X-ray measurements using several different single crystals. As a result, no atoms could be located in the channels of the X-ray refinement models for **1*α***. Thus, we concluded that remarkable disorder must exist for the ethanol molecules in the channels at this temperature.

The crystal structure of the second phase, **1*β***, which forms below the critical temperature, was determined by X-ray analysis at −40 °C by using the single crystal (**1*α***) that was prepared at room temperature. The space group *C*cc2 for **1*α*** was changed to the acentric space group *P*nc2 for **1*β***. In contrast to **1*α***, **1*β*** contained two crystallographically independent nickel centers, which yielded two types of one-dimensional chains that are made of equivalent nickel centers. The two chains are labeled Chain-A and Chain-B in Figure 2d. The inclinations of the two chains to the *a* and *b* axes are quite different to those of **1*α***; the tilting angles of the NO_3_—Ni—NO_3_ vectors to the *a* axis are about 35° for Chain-A and 0° (nearly parallel) for Chain-B. The phase transition accompanies a slide of the Chain-B (or Chain-A) of about 1 Å along the *c* axis. As a result, the coordinating nitrate anions are off-center above and below the square cavities of the two adjacent chains. The guest EtOH molecules, which were not structurally defined in **1*α***, were clearly observed in the channel-like cavities of **1*β*** The oxygen atom of the EtOH formed a weak hydrogen bond with an oxygen atom of a coordinating nitrate anion (O(4)—O(7) = 3.096; (2) Å).

**Scheme 3:**
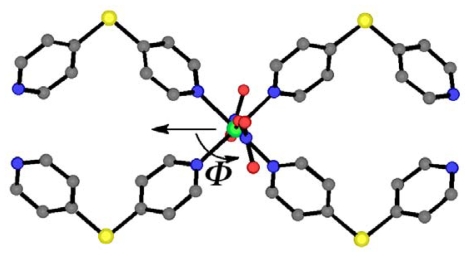
Definition of the *Φ* angle in the (**Ni**-**dps2**) chain.

The most significant effects of this phase transition on the porous structures are established by the rotation of the coordinating nitrate anions. When the angle of the NO_3_ plane of the coordinating anion to the channel direction, which is parallel to the *c* axis, is defined as *Φ* (Scheme 3), the angles of nitrate anions in **1*α*** are about 45° (and −45°). However, the *Φ* of nitrate anions in **1*β*** is about 80° (and −80°) for Chain-A, and 15° (and −15°) for Chain-B, respectively. That is, the planes of the nitrate anions of Chain-A are nearly perpendicular to the channel direction. The rotations jutted the nitrate anions into the channels, which resulted in the change of channel shape from “compressed hexagon” in **1*α*** to “T-shape” (5 × 2 + 2 × 3 Å) in **1*β***. The structural transformation narrowed the channel width from about 5 to 2 Å for the lower half of the channel window. This second phase with diminished channels is regarded as the *closed porous phase* induced by the temperature switch. In the previous communication, we showed that the including EtOH molecules were securely captured in the closed channels [6].

**Scheme 4:**
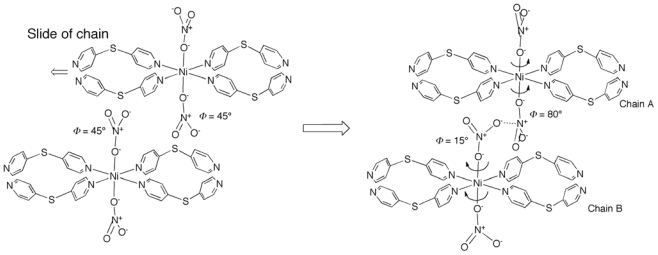
Plausible nitrate rotation mechanism. The slide of half of the chains make two nitrate anions in the adjacent chains closer, inducing rotations of nitrate anions by the N···O electrostatic interaction between the anions. As a result, half of the nitrate anions jut into the channels.

Weak electrostatic interactions are observed between the two nitrate anions in the adjacent chains; that is, oxygen atom (O(2)) of nitrate in Chain-B electrostatically interacts to nitrogen atom (N(6)) of nitrate in Chain-A. This result indicates that the rotations of nitrate anions are induced by the following mechanism (Scheme 4): the slide of half of the chains at the initial step makes two nitrate anions in the adjacent chains closer. The nitrate anions rotate to induce electrostatic interaction between N and O atoms of nitrate anions in the adjacent chains. As a result, nitrate anions in Chain B protrude into the channel-like cavities.

### 2.3. Crystal Structures of **2a** and **3**

The single crystal X-ray analysis data of satisfactory quality was not obtained for **2b**, despite several attempts of measurements due to cracking of the crystals occurring on heating. On the other hand, we have found that **2b** was isostrucural to [Co(dps)_2_(NO_3_)_2_] (**3**), which was prepared by treatment of Co(NO_3_)_2_·6H_2_O with dps in EtOH. Since the quality of the single crystal X-ray structure of **3** was better than **2b**, we mention the structure of **3** to explain that of **2b** here.

**Figure 3:**
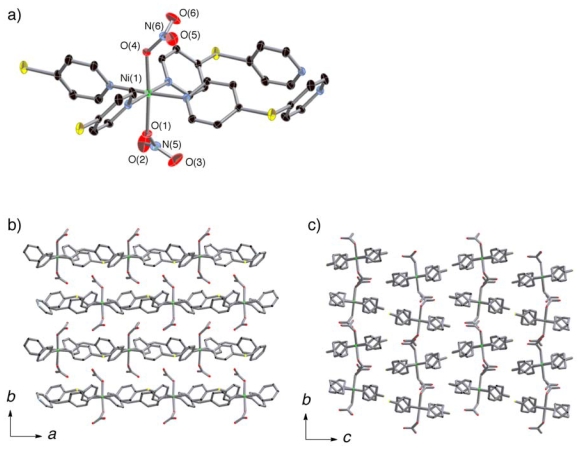
Coordination circumstance of Ni center of **2a** (**a**). Views of stacking aspect of (**Ni**-**dps2**) chains of **2a** in the *ab* plane (**b**), and assembled pattern of the chains along the *c* axis (**c**). Hydrogen atoms are omitted for clarity.

**Figure 4:**
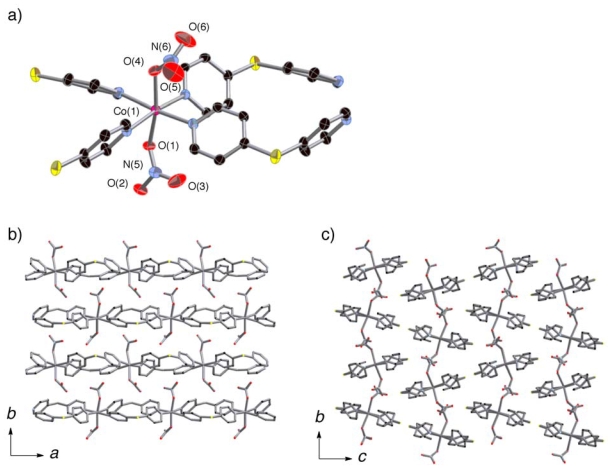
Coordination circumstance of Co center of **3** (**a**). Views of stacking aspect of (**Co**-**dps2**) chains of **3** in the *ab* plane (**b**), and assembled pattern of the chains along the *c* axis (**c**). Hydrogen atoms are omitted for clarity.

The coordination circumstances of **2a** and **3** (Figures 3a and 4a) were similar to that of **1*α***. Although **2a** and **3** were both guest-free coordination polymers formulated as [M(dps)_2_(NO_3_)_2_] (M = Ni, Co) constructed by (**Ni**-**dps2**) chains and [Co(dps)_2_(NO_3_)_2_] (**Co**-**dps2**) chains, their stacking patterns were not same (Figures 3 and 4). Their (**Ni/Co**-**dps2**) chains run along the *a* axis, and stack along the *b* axis. The *Φ* angles are about 52° for nitrate anions with N(5) atom and −74° for N(6) in **2b**, and the corresponding *Φ* angles are about 40° for nitrate anions with N(5) and −70° for N(6) in **3**. The inclinations of the chains to the *b* axis are smaller for **2a** compared to **3**; the tilting angles of the NO_3_—Ni—NO_3_ vectors to the *b* axis are about 6° for **2a** and 18° for **3**.

### 2.4. Rearrangement of (**Ni**-**dps2**) Chains by Guest Releases and Re-Inclusions

It is usually difficult to retain the structures of the flexible channel frameworks in the absence of guest molecules in the channels. Particularly, channels created among one-dimensional chains could be less stable because the frameworks are not supported three-dimensionally. Nevertheless, the dried compounds often adsorb the guest molecules and re-construct the initial structure. To understand the properties of the host frameworks of **1**, we characterized the release and re-inclusion properties of compound **1**.

**Figure 5:**
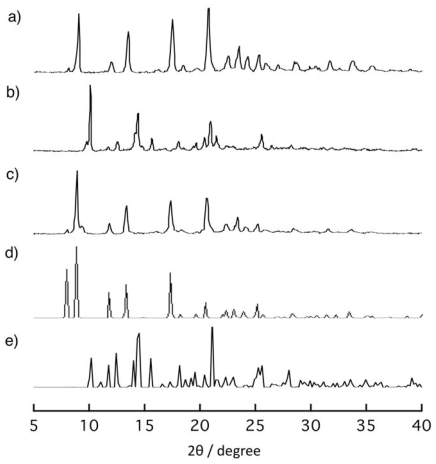
X-ray powder diffraction (XRPD) patterns of solid sample of **1** (**a**), its dried sample obtained on heating at 130 °C under reduced pressure (**b**), and the powder obtained by exposure of EtOH vapor to the dried sample (**c**) for three days. The simulation patterns based on the crystal structural analysis of **1*α*** (**d**) and **3** (**e**).

Figure 5 shows the changes of X-ray powder diffraction (XRPD) pattern of **1** responding to removals and re-inclusions of guest EtOH molecules. The XRPD pattern of **1** (Figure 5a) changed to a new one (Figure 5b) when it was dried on heating under reduced pressure. The XRPD pattern of the dried sample is consistent with that of the simulated XRPD pattern for **3** (Figure 5e). When the obtained dried sample was exposed to EtOH vapor for three days, the XRPD pattern of the initial powder was recovered (Figure 5c). This result clearly shows that the dried compound **2b** re-produced **1** by contact with EtOH vapor.

We reported that **1*β*** did not release EtOH molecules while **1*α*** released EtOH moleules in *m*-xylene [6]. The XRPD peaks of the powder sample obtained after the release of EtOH in *m*-xylene was rather consistent with that of **2a** than that of **3**, which is isostrucutral to **2b** (Supporting Information 1). This result means that **1** converted to **2a** by releasing guest EtOH molecules in *m*-xylene. On the other hand, exposure of EtOH vapor to **2a** did not produce **1** as studied by XRPD measurement (Supporting Information 2). These results reveal that the guest adsorption properties are not same between **2a** and **2b**.

### 2.5. Thermal Property of Ni-dps Compounds

Reaction of Ni(NO_3_)_2_·6H_2_O with dps in MeOH or acetone did not produce [Ni(dps)_2_(NO_3_)_2_] ·G (G = guest molecules) type coordination polymer, but yielded **2a** and **4. 4** does not have (**Ni**-**dps2**) chains, but shows one-dimensional coordination framework constructed by connection of Ni(II) centers by dps ligand. Interestingly, we found that the reaction in the mixed solution of MeOH/acetone (1:1) produced **5**, which is isostructural to **1*α***. The crystal structures of **4** and **5** are shown in Supporting Information. Although the positions of guest molecules in the channels were not determined due to the remarkable disorders, the result of elemental analysis implies the inclusions of MeOH and acetone (1:1) guest molecules per two Ni atoms.

**Figure 6:**
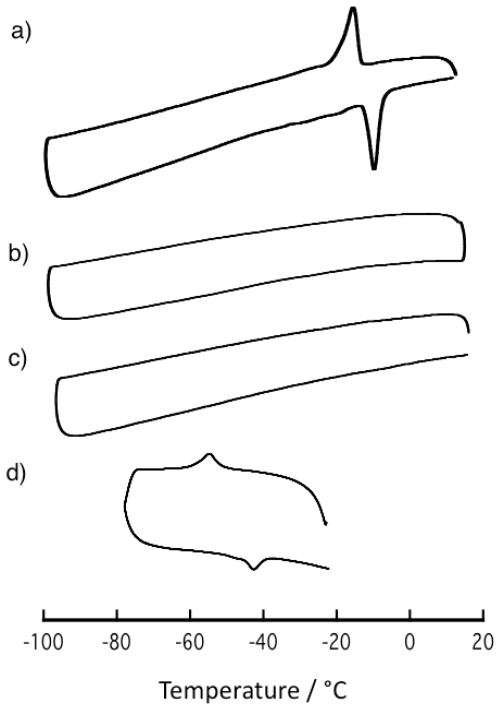
Differential scanning calorimeter (DSC) charts of **1** (**a**), **2a** (**b**), **2b** (**c**), and **5** (**d**).

While Differential scanning calorimeter (DSC) measurement revealed that **5** showed phase transition similar to **1** (Figure 6), the critical temperature (about −50 °C) is remarkably lower than that of **1**. In contrast to **1** and **5**, guest-free coordination polymers **2a** and **2b** did not show phase transition between −100 °C and 20 °C. This result means that the phase transition property is necessary for **1** type porous structure. Moreover, this result shows that kinds of guest molecules largely affect the critical temperature.

## 3. Experimental Section

### 3.1. Reagents and Materials

All reagents and solvents were purchased from commercial sources and were used as received. The thermal behavior was measured on Shimadzu DSC-60 differential scanning calorimeter (DSC) at a heating rate of 10 °C/min. Elemental analysis was performed on an analyzer Euro Vector EA 3000.

Synthesis of [Ni(dps)_2_(NO_3_)_2_] ·EtOH (**1**). An ethanol solution (25 mL) of dps (190 mg, 20 mmol) was allowed to diffuse into an ethanol solution (25 mL) of Ni(NO_3_)_2_·6H_2_O (290 mg, 20 mmol at room temperature. The obtained crystals were collected by filtration. Anal. Calcd for C_22_H_25_N_6_O_7_: C, 43.66; H, 3.66; N, 13.88. Found: C, 43.39; H, 3.49; N, 14.11.

Synthesis of [Ni(dps)_2_(NO_3_)_2_] (**2a**). A methanol solution (25 mL) of dps (190 mg, 20 mmol) was allowed to diffuse into an methanol solution (25 mL) of Ni(NO_3_)_2_·6H_2_O (290 mg, 20 mmol) at room temperature. The obtained crystals were collected by filtration. Anal. Calcd for C_20_H_16_N_6_NiO_6_S_2_: C, 42.96; H, 2.88; N, 15.03. Found: C, 42.51; H, 3.02; N, 14.99.

Synthesis of [Ni(dps)_2_(NO_3_)_2_] (**2b**). Single crystals of **1** were dried on heating at 130 °C under reduced pressure for 3 h. Anal. Calcd for C_20_H_16_N_6_NiO_6_S_2_: C, 42.96; H, 2.88; N, 15.03. Found: C, 42.85; H, 2.98; N, 14.86.

Synthesis of [Co(dps)_2_(NO_3_)_2_] (**3**). An ethanol solution (10 mL) of dps (190 mg, 1.0 mmol) was allowed to layer on the top of an ethanol solution (10 mL) of Co(NO_3_)_2_·4H_2_O (290 mg, 1.0 mmol). The solution was left for 1 month, giving pink crystals. Elemental analysis (%) calcd for C_20_H_16_CoN_6_O_6_S_2_: C, 42.94; H, 2.88; N, 15.02. Found: C, 42.29; H, 2.87;.N, 15.10.

Synthesis of [Ni(dps)(NO_3_)_2_(H_2_O)] ·Me_2_CO (**4**). An acetone solution (10 mL) of dps (190 mg, 1.0 mmol) was allowed to layer on the top of an acetone solution (10 mL) of Ni(NO_3_)_2_·6H_2_O (290 mg, 20 mmol) at room temperature. The solution was left for 1 month to yield blue crystals, which were collected by filtration. Anal. Calcd for C_13_H_16_N_4_NiO_8_S: C, 34.93; H, 3.61; N, 12.53. Found: C, 35.09; H, 3.65; N, 12.09.

Synthesis of [Ni(dps)_2_(NO_3_)_2_] ·0.5(MeOH·Me_2_CO) (**5**). An ethanol solution (10 mL) of dps (190 mg, 1.0 mmol) was allowed to layer on the top of an acetone solution (10 mL) of Ni(NO_3_)_2_·6H_2_O (290 mg, 20 mmol) at room temperature. The solution was left for 1 month, yielding blue crystals, which were collected by filtration. Anal. Calcd for C_22_H_21_N_6_NiO_7_S_2_: C, 43.73; H, 3.50; N, 13.91. Found: C, 43.03; H, 3.38; N, 13.54.

### 3.2. Crystal Structure Determinations

Each single crystal for X-ray analysis measurement was fixed on top of a glass fiber by epoxy glue (**1*β***, **2a**, **2b**, **3**), or sealed in a glass capillary with mother liquor (**1*α***, **4**, **5**). The data for all structures were measured on a Rigaku Mercury CCD system (MoK*α* radiation *λ* = 0.71073 Å). An empirical absorption correction was applied. The structures were solved by the direct method. Non-hydrogen atoms were refined anisotropically. Hydrogen atoms binding to carbon atoms were located on calculated positions, and were not refined but included. The crystallographic data of the compounds in this work is summarized in Table 1. Crystal structures of **1*α*** and **1*β***, which were reported in previous communication [6], were re-refined in this work to improve their analysis qualities.

## 4. Conclusions

Unique rearrangements of (**Ni**-**dps2**) chains of Ni-dps compounds have been studied. **1** showed two structural phases depending on the temperature. This compound mechanically *opens* and *closes* the channels. This dynamic structural change was caused by rotations of nitrate anions, which were induced by the slides of chains. **1** released guest EtOH molecules to yield **2a** when immersed in *m*-xylene, and to yield **2b** when heated at 130 °C under reduced pressure. While **2a** did not reproduce **1**, **2b** reproduced **1** by contact with EtOH vapor. The reaction of Ni(NO_3_)_2_·6H_2_O and dps did not produce porous frameworks with (**Ni**-**dps2**) chains in MeOH or acetone. Nevertheless, when the reaction was carried out in MeOH/acetone mixed solution, the dynamic porous framework isostructural to **1** was obtained. The existence of channel structures is necessary for the phase transition property responding to temperature variation in the Ni-dps system, and the critical temperature is largely affected by the including guest molecules. The further studies of the dynamic frameworks are in progress.

## Supporting Information

**Figure S1:**
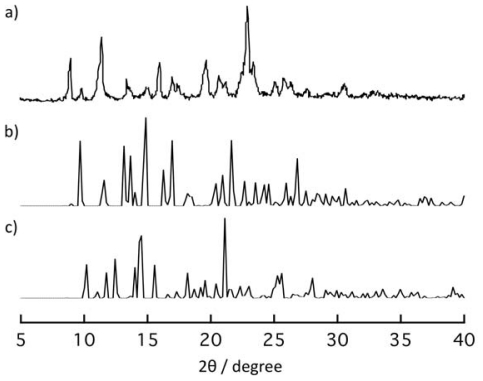
XRPD pattern after immersion of powder of **1*α*** in *m*-xylene for a few days (**a**) at room temperature. Simulation patterns of **2a** (**b**) and **3** (**c**). The XRPD pattern of (a) is not entirely consistent with that of (b) because of the effects of crystal morphology and the structural defects occurred during the structural transformation.

**Figure S2:**
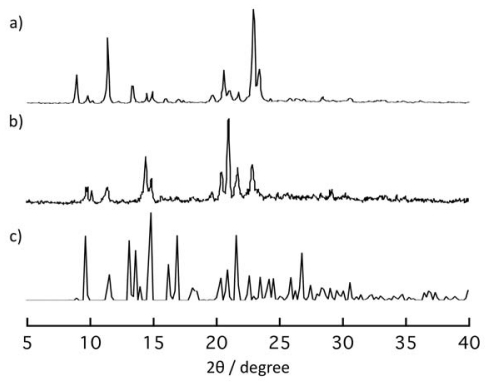
The XRPD pattern of powder sample of **2a** (**a**). The XRPD pattern of the powder sample of **2a** was exposed to EtOH vapor for three days (**b**). Simulation pattern of **2a** (**c**). The XRPD patterns between (a) and (b) are not entirely consistent because of the structural defects occurred during the structural transformation.

**Figure S3:**
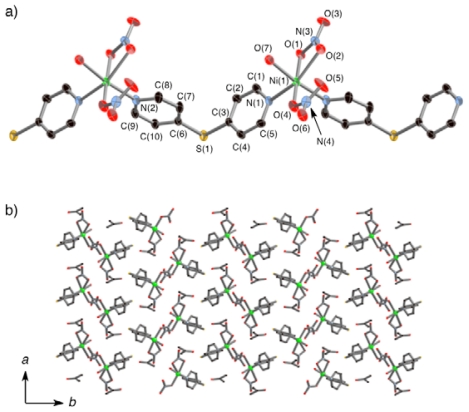
Crystal structure of **4**. The coordination circumstance (**a**) and stacking pattern in the *ab* plane (**b**).

**Figure S4:**
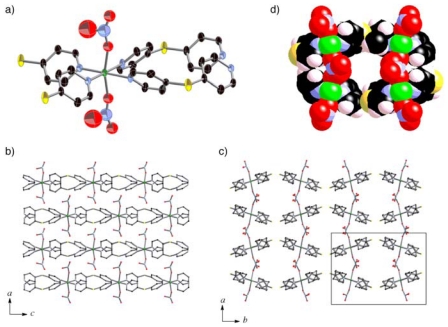
Crystal structure of **5**. The coordination circumstance (**a**) and stacking pattern in the *ac* plane (**b**) and *ab* plane (**c**). The channel structure with van der Waals radii is shown (**d**).

## Figures and Tables

**Table 1:** Crystallographic Data for **1*α***, **1*β***, **2a**, **2b**, **3**, **4**, and **5.**

Compound	**1*α***	**1*β***	**2a**	**2b**	**3**	**4**	**5**
Formula	C_22_H_22_N_6_O_7_NiS_2_	C_22_H_22_N_6_O_7_NiS_2_	C_20_H_22_N_6_O_7_NiS_2_	C_20_H_16_N_6_O_6_NiS_2_	C_20_H_16_CoN_6_O_6_S_2_	C_13_H_16_N_4_O_8_NiS	C_22_H_21_N_12_NiO_7_S_2_
Formula weight	605.27	605.27	559.20	559.20	559.44	447.05	604.26
Lattice	orthorhombic	orthorhombic	monoclinic	monoclinic	monoclinic	monoclinic	orthorhombic
*a*, Å	13.27(1)	13.1233(8)	10.016(4)	10.11(1)	10.200(1)	9.499(1)	13.20(2)
*b*, Å	19.88(2)	19.468(1)	12.554(5)	13.11(2)	12.9500(9)	21.841(2)	19.727(9)
*c*, Å	10.100(9)	10.1241(5)	18.960(9)	17.57(2)	17.4300(7)	10.293(2)	10.082(4)
*β*, °			107.921(6)	96.31(2)	95.790(2)	117.987(5)	
*V*, Å^3^	2665(3)	2586.6(2)	2268(1)	2314(4)	2290.6(3)	1885.8(4)	2611(7)
Space group	*Ccc2* (No. 37)	*P*nc2 (No. 30)	*P*2_1_/n (No. 14)	*P*2_1_/c (No. 14)	*P*2_1_/c (No. 14)	*P*2_1_/n (No. 14)	*Ccc2* (No. 37)
*Z*	4	4	4	4	4	4	4
*ρ* (calcd) g cm^−3^	1.508	1.554	1.637	1.605	1.622	1.574	1.529
*μ* (MoK*a*), mm^−1^	0.937	0.966	1.091	1.069	0.983	1.188	0.952
Radiation (*λ*, Å)	0.7107	0.7107	0.7107	0.7107	0.7107	0.7107	0.7107
Temperature (K)	298	233	298	298	298	298	298
Reflns collected	1969	20712	20623	6854	17163	14993	9423
Unique reflections	1641	5334	3171	2317	4294	3243	3237
Param refined	158	344	316	316	3116	244	131
*R* [*I* > 2σ (*I*)]	0.0774	0.0380	0.0591	0.243	0.0422	0.0443	0.0789
*R*_w_ [*I* > 2σ (*I*)]	0.1120	0.0557	0.0600	0.2878	0.0706	0.0529	0.1165
Goodness-of-fit	1.346	1.221	1.097	3.522	1.246	1.023	0.933

Reflns collected = Number of collected reflections, Param refined = Number of refined parameters, *R =* ∑||*F_o_*|−|*F_c_*||/∑|*F_o_*|, *R_w_* *=* [∑ω(|F_o_|−|F_c_|)^2^_/∑ ω_|F_o_|^2^)]^1/2^
